# Non-right-handedness in children born extremely preterm: Relation to early neuroimaging and long-term neurodevelopment

**DOI:** 10.1371/journal.pone.0235311

**Published:** 2020-07-06

**Authors:** Alise A. van Heerwaarde, Laura T. van der Kamp, Niek E. van der Aa, Linda S. de Vries, Floris Groenendaal, Marian J. Jongmans, Rian J. C. Eijsermans, Corine Koopman-Esseboom, Inge-Lot C. van Haastert, Manon J. N. L. Benders, Jeroen Dudink

**Affiliations:** 1 Department of Neonatology, Wilhelmina Children’s Hospital, University Medical Centre Utrecht, Utrecht University, Utrecht, The Netherlands; 2 Department of Paediatric Psychology, Wilhelmina Children's Hospital, University Medical Centre Utrecht, Utrecht University, Utrecht, The Netherlands; 3 Department of Paediatric Physical Therapy and Exercise Physiology, Wilhelmina Children's Hospital, University Medical Centre Utrecht, Utrecht University, Utrecht, The Netherlands; Western University, CANADA

## Abstract

**Objective:**

This study aimed to define the prevalence and predictors of non-right-handedness and its link to long-term neurodevelopmental outcome and early neuroimaging in a cohort of children born extremely preterm (<28 weeks gestation).

**Methods:**

179 children born extremely preterm admitted to the Neonatal Intensive Care Unit of our tertiary centre from 2006–2013 were included in a prospective longitudinal cohort study. Collected data included perinatal data, demographic characteristics, neurodevelopmental outcome measured by the Bayley Scales of Infant and Toddler Development at 2 years and the Movement Assessment Battery for Children at 5 years, and handedness measured at school age (4–8 years). Magnetic resonance imaging performed at term-equivalent age was used to study overt brain injury. Diffusion tensor imaging scans were analysed using tract-based spatial statistics to assess white matter microstructure in relation to handedness and neurodevelopmental outcome.

**Results:**

The prevalence of non-right-handedness in our cohort was 22.9%, compared to 12% in the general population. Weaker fine motor skills at 2 years and paternal non-right-handedness were significantly associated with non-right-handedness. Both overt brain injury and fractional anisotropy of white matter structures on diffusion tensor images were not related to handedness. Fractional anisotropy measurements showed significant associations with neurodevelopmental outcome.

**Conclusions:**

Our data show that non-right-handedness in children born extremely preterm occurs almost twice as frequently as in the general population. In the studied population, non-right-handedness is associated with weaker fine motor skills and paternal non-right-handedness, but not with overt brain injury or microstructural brain development on early magnetic resonance imaging.

## Introduction

In Europe, preterm birth accounts for 5.5–11.1% of all live births (2008) [1]. Health care for these vulnerable children improved over the past decades and survival chances are still increasing. Compared to children born term, children born preterm have a higher risk of abnormal neurodevelopment [2]. Interestingly, one of the less obvious differences between preterm and term born children is the higher prevalence of non-right-handedness (NRH), a combination of left- and mixed-handedness. The underlying mechanism(s), however, remain poorly understood [3].

Different hypotheses explaining the underlying mechanisms of the increased NRH prevalence in children born preterm have been proposed: genetic predisposition, brain pathology, or a combination of both [3]. Studies aiming to test these theories have suggested multiple demographic and perinatal factors to be related to NRH, although they are all surrounded by some inconsistency. Examples are gestational age (GA), birth weight, multiple birth, maternal age, reported birth stress, birth season, Apgar scores, and the presence and side of overt brain injury. Also the factors sex and parental handedness have been suggested to be associated with NRH in both the general and ex-preterm populations [4–12].

Currently advanced imaging techniques can provide new insights in the relation between NRH and microstructural brain changes. A relationship was demonstrated between the microstructural integrity of the corpus callosum (CC) in children born very preterm (<32 weeks gestation) and handedness, using diffusion tensor imaging (DTI) scans [12]. To our knowledge, this has been the only DTI study exploring handedness in children born preterm and no studies in extremely preterm (EPT; [<28 weeks gestation]) cohorts have yet been conducted.

In addition, NRH in (very) preterm infants has been associated with atypical and poorer cognitive and motor development [12,13]. A recent study by Campbell et al. found that term born infants with a late developing right-hand preference are significantly less developed on neuromotor measures than infants with no preference, early right preference, and left-hand preference infants at 6 months of age [14]. However, another recent study by Burnett et al. reported equal cognitive, academic, motor and behavioural performance among left- and right-handers in very preterm and EPT populations, but reported that mixed-handed children show greater odds of functional deficits across these domains [15].

Thus, although repeatedly described, the cause of the higher NRH prevalence, the predicting factors, the possible association with neurodevelopment and the link with early neuroimaging findings are still unclear. Therefore, the aim of our study was to determine the prevalence of NRH in EPT children assessed at school age and the demographic and perinatal factors associated with NRH in this population. Furthermore, the association between handedness and macro- and microstructural brain findings on neuroimaging and neurodevelopmental outcome of these children was studied. In order to evaluate the possible effect of microstructural brain changes on NRH, DTI was used [16].

We hypothesized that the prevalence of NRH in EPT born children would be higher than in the general population (>12%) [3]. Additionally, we assumed this would be associated with (macro- and microstructural) brain difference seen on term magnetic resonance imaging (MRI)/DTI scans and poorer neurodevelopmental outcome at 2 years corrected age (CA) and school age (4–8 years).

## Methods

### Participants

All EPT children admitted to our Neonatal Intensive Care Unit between September 2006 and October 2013, who were consecutively enrolled in previous prospective, longitudinal studies, were eligible for our study. The original studies were approved by the Medical Research Ethics Committee of University Medical Centre Utrecht. Informed parental consent was obtained for inclusion in the previous cohort studies. No additional informed consent was required for the current handedness study, according to the Medical Research Ethics Committee. Neurodevelopmental outcome was assessed at multiple time points as part of routine follow-up, including 2 years CA and school age (4–8 years). The availability of handedness data as assessed at school age (4–8 years) during the child’s assessment with the Movement Assessment Battery for Children second edition, Dutch version (MABC-2-NL), was defined as an inclusion criterion. Therefore, children who did not undergo the MABC-2-NL were excluded. Among those were children with cerebral palsy or Erb’s palsy, as they could not be tested according to the MABC-2-NL guidelines [17]. For DTI sub-analysis, subjects were eligible if a DTI scan was performed at term-equivalent age (TEA). Exclusion criteria were poor quality and presence of scanning artefacts, congenital anomalies and severe brain injury that affected the registration of the scans (e.g. severe intraventricular haemorrhage [IVH] grade 4, severe stroke, severe post-haemorrhagic ventricular dilatation [PHVD]).

### Variables

The following characteristics were collected from the child’s medical file: sex, date of birth, GA at birth, birth weight, plurality, birth season, Apgar scores at 1, 5 and 10 minutes and postmenstrual age (PMA) at MRI scan. Birth seasons were defined as starting at the 21^st^ of December, March, June and September. Parental handedness as reported by parents was noted during the child’s neurodevelopment assessment at 2 years CA and school age (4–8 years). Data on maternal and paternal education level were obtained during the two-year follow-up (highest education level, reported as low, middle or high, according to the classification of Statistics Netherlands [Statistics Netherlands, The Hague, The Netherlands; http://www.cbs.nl/en-GB/menu/home/default.htm]).

As a standard clinical care procedure, MRI was performed with parental permission around TEA on a 3.0 T MR system (Achieva, Philips Medical System, Best, The Netherlands), using a Philips SENSE head coil, in all clinically stable neonates. The routine scanning protocol consisted of both conventional sequences (e.g. T1- and T2-weighted images) and diffusion weighted sequences [18,19]. Additionally, DTI scans were acquired from 2008 onwards in the axial plane with one non-diffusion weighted image and 32 diffusion weighted images in 32 non-collinear directions (b-value 800 s/mm^2^). The protocol used was as follows: echoplanar imaging factor 55, TR/TE 5685/70 ms, field of view 180 × 146 mm, acquisition matrix 128 × 102 mm, reconstruction matrix 128 × 128 mm, 50 slices with 2 mm thickness without gap, total scan time 4.4 min.

All scans were assessed by an experienced paediatric radiologist and two experienced neuro-neonatologists (LdV, FG) on neonatal brain injury. Additionally, LdV and FG assessed the MRI scans using a previously described MRI scoring system for brain injury by Kidokoro et al. [20]. Reports of neonatologist and radiologist regarding focal brain injury were compared, and in case of disagreement, scans were re-evaluated by an experienced neuro-neonatologist (JD). DTI scans were visually assessed and excluded in case of bad quality, motion or scanning artefacts leading to reduced assessability [19]. Consensus on scoring was reached in all cases. Brain injury was classified as: (1) focal brain injury (IVH, cerebellar haemorrhage[(CBH] and stroke) and (2) global brain injury (PHVD and moderate or severe global score according to the Kidokoro scoring tool) [20]. Both IVH and CBH were recorded as the highest grade on neonatal ultrasound (if cerebellum was assessed), routine MRI at 30 weeks and routine MRI at 40 weeks PMA. IVH was classified according to the IVH grading system of Papile et al. [21]. CBH was grouped as no or <6 punctate lesions, ≥6 punctate lesions, large unilateral bleeding, and large bilateral bleeding with or without vermis involvement. PHVD was defined as a ventricular index >97th percentile according to Levene [22].

Neurodevelopmental outcome was assessed as part of routine follow up, using the Bayley Scales of Infant and Toddler Development 3^rd^ version (BSITD-III-NL) at 24–30 months CA and the MABC-2-NL at an uncorrected age of 50 up to 99 months [17,23]. The composite score for cognition and the scaled scores for fine and gross motor on the BSITD-III-NL were calculated and corrected for PMA at examination to avoid bias. The language subtest was not assessed; due to the limited time children are able to concentrate during one session. Scores were provided as standard scores on the MABC-2-NL. All included study participants for analysis completed the two subtests of the BSITD-III-NL and the three subtests of the MABC-2-NL. The children were tested by either a single developmental specialist or a single child psychologist, depending on the year of assessment.

Hand preference was assessed by a trained paediatric physical therapist during the MABC-2-NL at school age (4–8 years). The assumption of a relatively stable hand preference at this age was concordant with existing literature [24]. Hand preference was assessed by a trained paediatric physical therapist during the MABC-2-NL at school age (4–8 years). The assumption of a relatively stable hand preference at this age was concordant with existing literature [24]. According to the MABC-2-NL manual, the preferred hand is “the hand used to write or draw with”. Before beginning the test, the child should be asked to write its name on a separate piece of paper or draw a picture. Secondly, a coin was placed on the midline of the child and the hand used to pick it up. The combination of both defined the preferred hand. This could be left, right or indecisive/mixed, in case of a discrepancy between the hand used for writing and the hand used for picking up the coin. Parents were asked if the observed hand dominance was in accordance with the child’s preference noted at home during writing or drawing. In case of disagreement, handedness was scored as indecisive/mixed. Since the purpose of this study was to address NRH, left-handed and indecisive/mixed-handed children were combined in one category for the analyses.

The preferred hand was rechecked during other subtests of the MABC-2-NL. These subtests are the putting coins in a money box, bicycle trail and throwing a seed bag. These tasks were not decisive for the final hand preference, but could change it to indecisive/mixed in case of frequent disagreement between all different tests.

In the “putting coins in a box” task, both hands were alternately observed when putting coins in a box. The preferred hand is the one performing the task faster and more fluently. According to the manuals, time recording in the putting-coins-in-a-box tests was started “when the free hand leaves the mat”. The other hand holds the box. For the studied age category, for each hand there was a practice attempt using six coins. The bicycle trail is a tracing assignment of the MABC-2-NL, in which the child traces a bicycle trail using a pencil. The preferred hand is the one to write with. In throwing a seed bag, the hand used to throw with is noted as the preferred hand. According to the manual, using both hands does not change the hand preference to indecisive/mixed. Further details on how the specific tests were performed, can be written in the MABC-2-NL manual [17].

### DTI data analysis

DTI data were processed using the diffusion MR toolbox ‘ExploreDTI’ [25]. The diffusion-weighted images were realigned to the b0-image to correct for subject motion and eddy current induced geometric distortions, in which the diffusion gradients were adjusted with the proper b-matrix rotation. The skull was removed from the b0 images using the Brain Extraction Tool. Next, the diffusion tensor was fitted for each voxel using a nonlinear least squares method.

The tensor was exported for further analyses using the Diffusion Tensor Imaging ToolKit (DTI-TK). DTI-TK allows tensor-based registration and normalization of DTI data [26]. DTI-TK was used to create a DTI for subsequent registration of the individual tensor data to the templates. The template was based on 19 DTI scans of infants with no brain injury. To create the initial template, the tensor images were rotated to the same orientation by a rigid alignment and averaged using a Log-Euclidean mean. The template was iteratively optimized, initially using rigid, followed by affine alignments and finally non-linear alignments. After finishing the template, the tensor data of all subjects were registered to the corresponding templates using the same rigid, affine and non-linear alignment.

After registration of all data, individual fractional anisotropy (FA) maps were exported for tract-based spatial statistics (TBSS), part of the FMRIB software library [27]. The aligned images were used to create an average FA map. This map was thinned to generate a mean FA skeleton, which represents the centre of all white matter tracts common in the aligned FA images. The skeleton was thresholded at 0.15 and individual FA data were projected on the skeleton. Voxelwise cross-subject statistics was performed using Randomise (v2.5) using univariate linear modelling. The relation between FA and handedness, BSITD-III-NL scores and MABC-2-NL scores was studied, while correcting for PMA and GA at birth. The results were corrected comparison by controlling for the family-wise error rate following threshold-free cluster enhancement and are shown at p< 0.05.

### Statistical analyses

Data were analysed using SPSS version 24 (SPSS Inc., Chicago, Illinois, USA). Based on NRH prevalences described in literature (12% in children born term; 22% in children born very preterm), the required sample size to detect a difference in prevalence compared to the general population was 98, with an alpha of 0.05 and a desired power of 0.8 [3]. The assumption of normal distribution was checked for all variables. Differences in demographic, perinatal factors, brain injury and neurodevelopmental outcome measures between both handedness groups, were assessed using a two-sample unpaired t-test, a Mann-Whitney-U-test, a two-sided chi-square test or Fisher’s exact test where appropriate. Linear regression with the neurodevelopmental outcome measures as dependent variables were performed to control for possible confounders for the interaction between handedness and neurodevelopmental outcome: sex, birth weight <-1 SD and maternal education level. Since BSITD-III-NL scores were already corrected for PMA at examination and both BSITD-III-NL scores and MABC-2-NL scores for age at assessment date, adjusting for these variables was not considered necessary. Additionally, multivariable logistic regression was performed with handedness as the dependent variable. The largest set of variables without multicollinearity was used, and variables were excluded backward based on the highest p-value. For all analyses, p-values <0.05 were considered significant.

## Results

### Participants

**Fig 1:**
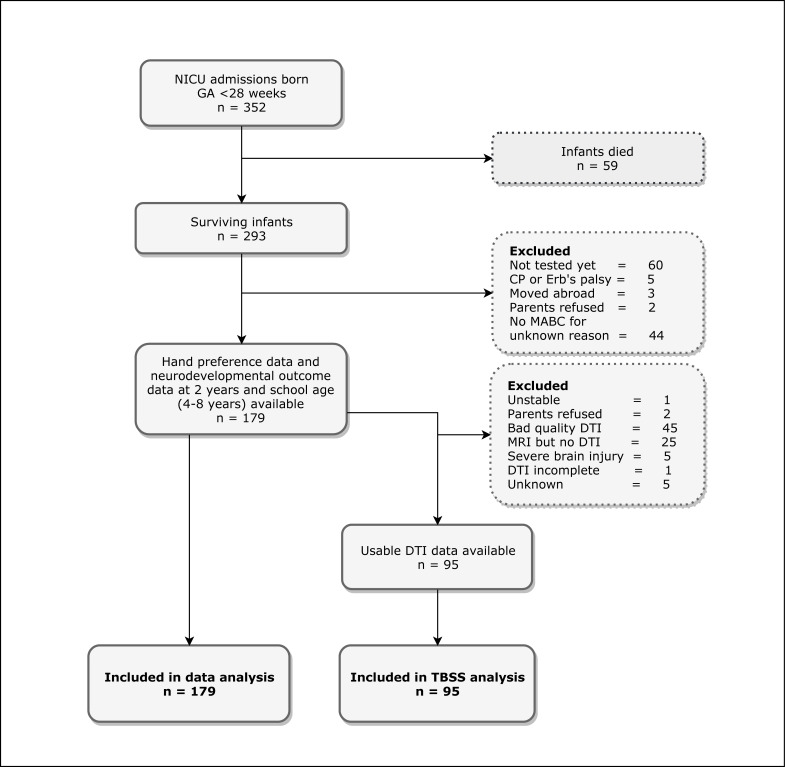
Flowchart of study participants. Abbreviations: NICU, Neonatal Intensive Care Unit; GA, gestational age; CP, cerebral palsy; MABC-2-NL, Movement Assessment Battery for Children, second edition, Dutch version; DTI, diffusion tensor imaging; MRI, magnetic resonance imaging; TBSS, tract-based spatial statistics.

The total number of eligible patients was 352, of whom 59 died in the neonatal period. For 179 EPT children, data on hand preference could be obtained during the MABC-2-NL. For all of them, neurodevelopmental outcome data at 2 years CA and school age (4–8 years) were available. DTI scans were usable for TBSS analysis in 95 cases. An overview of the in- and excluded patients and reasons for exclusion is presented in Fig 1.

### NRH and demographic and perinatal measures

In Table 1, baseline characteristics of the study population are presented. All groups of variables with missings, used for sub-analyses, did not differ significantly from the whole population on any variable.

**Table 1:** Perinatal and demographic characteristics of all included children, categorised by hand preference.

		Total	NRH	RH	p*	Cramér's *V*
		(n = 179)	(n = 41)	(n = 138)		
**Demographic and perinatal measures**						
NRH	n (%)	41 (22.9)	NA	NA	NA	
Left-handedness	n (%)	35 (19.6)	35 (85.4)	NA	NA	
Mixed-handedness	n (%)	6 (3.4)	6 (14.6)	NA	NA	
Sex	Male, n (%)	87 (48.6)	23 (56.1)	64 (46.4)	0.274	
Gestational age (weeks)	Mean (SD)	26.5 (1.0)	26.4 (0.9)	26.5 (1.0)	0.262	
Birth weight (g)	Mean (SD)	886 (163)	842 (142)	899 (167)	0.350	
Plurality	Singleton, n (%)	119 (66.5)	31 (75.6)	88 (63.8)	0.158	
Birth season	Winter, n (%)	51 (28.5)	13 (31.7)	38 (27.5)	0.492	
	Spring, n (%)	38 (21.2)	6 (14.6)	32 (23.2)		
	Summer, n (%)	45 (25.1)	13 (31.7)	32 (23.2)		
	Autumn, n (%)	45 (25.1)	9 (22.0)	36 (26.1)		
Apgar score at 1 minute^1^	Median (IQR)	5 (2)	4.5 (3)	5 (3)	0.600	
Apgar score at 5 minutes^1^	Median (IQR)	7 (2)	7 (2)	7 (2)	0.961	
Apgar score at 10 minutes^2^	Median (IQR)	8 (1)	9 (2)	8 (1)	0.690	
Post-menstrual age in days at MRI scan at term equivalent age^3^	Median (IQR)	41.1 (0.6)	41.3 (0.5)	41.1 (0.8)	0.105	
Age in months at MABC-2-NL	Median (IQR)	70.1 (1.8)	70.1 (2.5)	70.1 (1.6)	0.130	
**Scanning data**						
MRI at term equivalent age available	n (%)	171 (95.5)	40 (97.6)	131 (94.9)	0.684	
Kidokoro score available	n (%)	167 (93.3)	40 (97.6)	127 (92.0)	0.301	
DTI usability	n (%)	95 (53.1)	27 (65.9)	68 (49.3)	0.062	
**Parental measures**						
Hand preference mother^4^	NRH, n (%)	28 (17.4)	8 (20.0)	20 (16.5)	0.616	
Hand preference father^5^	NRH, n (%)	29 (18.4)	12 (31.6)	17 (14.2)	**0.016**	0.192
Parental NRH^5^	At least one parent NRH, n (%)	52 (32.9)	18 (47.4)	34 (28.3)	**0.030**	0.173
Maternal education level^6^	Low, n (%)	40 (22.9)	9 (22.0)	31 (23.1)	0.801	
	Middle, n (%)	65 (37.1)	17 (41.5)	48 (35.8)		
	High, n (%)	39.1 (40.0)	15 (36.6)	55 (41.0)		
Paternal education level^7^	Low, n (%)	27 (25.2)	6 (25.0)	21 (25.3)	0.444	
	Middle, n (%)	34 (31.8)	10 (41.7)	24 (28.9)		
	High, n (%)	46 (43.0)	8 (33.3)	38 (45.8)		
**Brain inury measures**						
Combined brain injury^8^	n (%)	81 (47.4)	15 (37.5)	66 (50.4)	0.153	
Focal lesions total^9^	n (%)	75 (43.6)	15 (37.5)	60 (45.5)	0.374	
IVH	n (%)	72 (40.2)	15 (36.6)	57 (41.3)	0.588	
CBH^8^	n (%)	11 (6.4)	3 (7.5)	8 (6.1)	0.720	
Global lesions total^10^	n (%)	25 (15.0)	4 (10.0)	21 (16.5)	0.312	
PHVD	Surgical intervention n (%)	9 (5.0)	2 (4.9)	7 (5.1)	1.000	
Kidokoro global score^10^	Moderate or severe, n (%)	21 (12.6)	4 (10.0)	17 (13.4)	0.573	
Kidokoro white matter score^10^	Moderate or severe, n (%)	30 (18.0)	5 (12.5)	25 (19.7)	0.302	
Kidokoro gray matter score^10^	Moderate or severe, n (%)	36 (21.6)	13 (32.5)	23 (18.1)	0.054	
Kidokoro deep gray matter score^10^	Moderate or severe, n (%)	2 (1.2)	1 (2.5)	1 (0.8)	0.423	
Kidokoro cerebellar score^10^	Moderate or severe, n (%)	19 (11.4)	2 (5.0)	17 (13.4)	0.251	

Abbreviations: NRH, non-right-handedness; RH, right-handedness; NA, not applicable; SD, standard deviation; IQR, interquartile range; MABC-2-NL, Movement Assessment Battery for Children second edition, Dutch version; MRI, magnetic resonance imaging; DTI, diffusion tensor imaging; IVH, intraventriculair haemorrhage; CBH, cerebellar haemorrhage; PHVD, post-haemorrhagic ventricular dilatation.

For some variables, the total sample size is smaller than the reported original sample due to missing values: ^1^ n = 177, ^2^ n = 95, ^3^ n = 171, ^4^ n = 161, ^5^ n = 158, ^6^ n = 175, ^7^ n = 107, ^8^ n = 171, ^9^ n = 172, ^10^ n = 167.

*Independent samples t-test for continuous variables; Pearson Chi-Square (χ^2^) for categorical variables; Fisher's Exact Test for dichotomous variables with expected cell counts <5.

A prevalence of NRH of 22.9% was found in the overall study population, consisting of 19.6% left-handers and 3.4% mixed-handers. For none of the other demographic and perinatal measures, a statistically significant difference was found.

The majority of the included children had an MRI at TEA (95.5%) on which the Kidokoro scoring tool was applied in 97.7% of the cases. Usable DTI scans were available for 53.1% of the included children.

Regarding the parental measures, both the presence of at least one parent with NRH and paternal NRH were observed more frequently in the NRH group (p = 0.030; p = 0.016), although effect sizes were limited (Cramér’s *V* = 0.192; Cramér’s *V* = 0.173). However, maternal NRH did not differ significantly between the two groups (p = 0.616).

### Brain injury measures

81 children (47.4%) were diagnosed with at least one type of brain lesion which was more common among right-handed children (p = 0.153). This was mainly due to the higher percentage of IVH in the right-handed group, since CBH and global lesions were seen more often in the NRH group. 43.6% of the children had one or more focal lesions and 15.0% was classified as having global brain lesions. Table 2 provides a more detailed overview of the prevalence of every sub-category of brain injury and the affected side. As shown, no correlation between the side of any type of lesion and hand preference could be detected.

**Table 2:** Brain injury measures of all included children, categorised by hand preference.

			Total	NRH	RH	*p*
			(nᵃ = 179)	(nᵃ = 41)	(nᵃ = 138)	
Combined brain injury^1^	n (%)		81 (47.4)	15 (37.5)	66 (50.4)	0.153
Focal lesions total^2^	n (%)	Total	75 (43.6)	15 (37.5)	60 (45.5)	0.374
		L or L>R	27 (15.7)	5 (12.5)	22 (16.7)	1.000
		R or L<R	15 (8.7)	3 (7.5)	12 (9.1)	
		L = R	33 (19.2)	7 (17.5)	26 (19.7)	
IVH grade	IVH total	Total	72 (40.2)	15 (36.6)	57 (41.3)	0.588
		L or L>R	27 (15.1)	5 (12.2)	22 (15.9)	0.931
		R or L<R	15 (8.4)	3 (7.3)	12 (8.7)	
		L = R	30 (16.8)	7 (17.1)	23 (16.7)	
	IVH grade 1 or 2, n (%)	Total	63 (35.2)	13 (31.7)	50 (36.2)	0.594
		L or L>R	23 (12.8)	4 (9.8)	19 (13.8)	0.920
		R or L<R	12 (6.7)	3 (7.3)	9 (6.5)	
		L = R	28 (15.6)	6 (14.6)	22 (15.9)	
	IVH grade 3 or 4, n (%)	Total	9 (5.0)	2 (4.9)	7 (5.1)	1.000
		L or L>R	4 (2.2)	1 (2.4)	3 (2.2)	0.667
		R or L<R	3 (1.7)	0 (0.0)	3 (2.2)	
		L = R	2 (1.1)	1 (2.4)	1 (0.7)	
CBH severity^1^	CBH total	Total	11 (6.4)	3 (7.5)	8 (6.1)	0.720
		L or L>R	1 (0.6)	1 (2.5)	0 (0.0)	0.055
		R or L<R	1 (0.6)	1 (2.5)	0 (0.0)	
		L = R	9 (5.3)	1 (2.5)	8 (6.1)	
	≥6 punctate lesions, n (%)	L = R	8 (4.7)	1 (2.5)	7 (5.3)	0.683
	Large unilateral bleeding, n (%)	Total	2 (1.2)	2 (5.0)	0 (0.0)	0.054
		L or L>R	1 (0.6)	1 (2.5)	0 (0.0)	1.000
		R or L<R	1 (0.6)	1 (2.5)	0 (0.0)	
	Large bilateral bleeding, with or without vermis involvement, n (%)	L = R	1 (0.6)	0 (0.0)	1 (0.8)	1.000
Global lesions total^3^	n (%)		25 (15.0)	4 (10.0)	21 (16.5)	0.312
PHVD	Surgical intervention, n (%)		9 (5.0)	2 (4.9)	7 (5.1)	1.000
Kidokoro global score^3^	No or mild, n (%)		146 (87.4)	36 (90.0)	110 (86.6)	0.629
	Moderate, n (%)		18 (10.8)	3 (7.5)	15 (11.8)	
	Severe, n (%)		3 (1.8)	1 (2.5)	2 (1.6)	
Kidokoro white matter score^3^	No or mild, n (%)		137 (82.0)	35 (87.5)	102 (80.3)	0.481
	Moderate, n (%)		23 (13.8)	3 (7.5)	20 (15.7)	
	Severe, n (%)		7 (4.2)	2 (5.0)	5 (3.9)	
Kidokoro gray matter score^3^	No or mild, n (%)		131 (78.4)	27 (67.5)	104 (81.9)	0.138
	Moderate, n (%)		24 (14.4)	8 (20.0)	16 (12.6)	
	Severe, n (%)		12 (7.2)	5 (12.5)	7 (5.5)	
Kidokoro deep gray matter score^3^	No or mild, n (%)		165 (98.8)	39 (97.5)	126 (99.2)	0.423
	Moderate, n (%)		1 (0.6)	1 (2.5)	0 (0.0)	
	Severe, n (%)		1 (0.6)	0 (0.0)	1 (0.8)	
Kidokoro cerebellar score^3^	No or mild, n (%)		148 (88.6)	38 (95.0)	110 (86.6)	0.064
	Moderate, n (%)		13 (7.8)	0 (0.0)	13 (10.2)	
	Severe, n (%)		6 (3.6)	2 (5.0)	4 (3.1)	

Abbreviations: NRH, non-right-handedness; RH, right-handedness; IVH, intraventriculair haemorrhage; CBH, cerebellar haemorrhage; PHVD, post-haemorrhagic ventricular dilatation; L, left; R, right.

For some variables, the total sample size is smaller than the reported original sample due to missing values: ^1^ n = 171, ^2^ n = 172, ^3^ n = 167.

### NRH and neurodevelopmental outcome measures

Univariate analysis of all neurodevelopmental outcome subtests revealed a significant association between lower fine motor scaled score on the BSITD-III-NL and NRH (Table 3, p = 0.008), also after adjusting for pre-defined possible confounders as indicated in Table 3 (p = 0.016). In both cases, effect sizes were small (*r* = 0.198; *r* = 0.181). Of all other neurodevelopmental outcome variables, no significant association with handedness was found. Of note is that children born EPT of both categories obtained significantly lower scores in all three MABC-2-NL domains than the general population. In all domains together (total standard score), 37.1% of the right-handers and 48.4% of the non-right-handers had an abnormal score (≤6 percentile), compared to 5.0% in the general population. 26.1% of the right-handers and 24.4% of the non-right-handers had a borderline score (7–16 percentile), compared to 10.0% in the general population.

**Table 3:** Neurodevelopmental outcome of all included children, categorised by hand preference.

	Total	NRH	RH	*p* (unadjusted)	*r* (unadjusted)	*p* (adjusted)*	*r* (adjusted)*
	(n = 179)	(n = 41)	(n = 138)		* *		
**Dutch BSITD-III**	**Median (IQR)**	**Median (IQR)**	**Median (IQR)**				
Cognitive composite score	101 (19)	101 (19)	101 (24)	0.488		0.890	
Fine motor scaled score	13 (3)	12 (3)	13 (3)	**0.008**	0.198	**0.016**	0.181
Gross motor scaled score	10 (3)	9 (3)	10 (3)	0.073		0.255	
**MABC-2-NL**	**Median (IQR)**	**Median (IQR)**	**Median (IQR)**	** **	** **	** **	** **
Total standard score	6 (3)	6 (4)	7 (3)	0.392		0.843	
Manual dexterity standard score	7 (3)	6 (3)	7 (3)	0.104		0.274	
Catching and aiming standard score	8 (4)	8 (3)	8 (4)	0.583		0.749	
Balance standard score	8 (3)	8 (3.5)	8 (3.25)	0.778		0.161	

Abbreviations: NRH, non-right-handedness; RH, right-handedness; IQR, interquartile range; BSITD-III, Bayley Score of Infant and Toddler Development, third edition; MABC-2-NL, Movement Assessment Battery for Children, second edition, Dutch version.

* Adjusted for sex, birth weight <-1 SD and maternal education level. Sample size was smaller (n = 175) due to missing values of maternal education level.

### Logistic regression

Subsequent logistic regression analysis yielded a model of two variables in relation to child handedness: hand preference of the father and the BSITD-III fine motor scaled score. Univariate analysis results, as described above, already determined these two variables as the significantly related factors, and they remained significant after considering the influence of other factors (p = 0.039 and p = 0.019, respectively). The corresponding odds ratios (OR) were 0.399 (95% confidence interval (CI) [0.166, 0.956]) and 0.820 (95% CI [0.695, 0.968]), respectively.

### TBSS analysis

**Fig 2:**
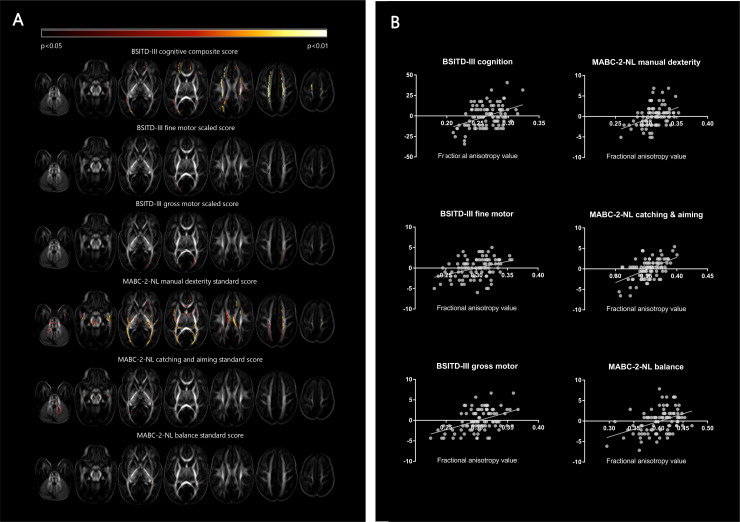
Tract-based spatial statistics: White matter changes on diffusion tensor imaging in relation to neurodevelopmental outcome. The results of the tract-based spatial statistics analysis. A. The color indicates the degree of significance of the association of its fractional anisotropy (FA) value with the studied outcome variable, as presented at the top of each series of images. B. Partial regression plots showing the linear relationship between the different outcome measures and the FA value extracted from the significant voxels, corrected for GA at birth and PMA at scan. For each subtest a significant positive correlation between FA and neurodevelopmental outcome is shown. Abbreviations: BSITD-III, Bayley Scales of Infant and Toddler Development, third edition, Dutch version; MABC-2-NL, Movement Assessment Battery for Children, second edition, Dutch version; FA, fractional anisotropy.

Fig 2 is a graphical representation of the TBSS analysis results. Since hand preference (both right- versus left-handedness and right- versus non-right-handedness) was not found to be associated with the FA values of any white matter tract, the corresponding brain images are not shown.

Extensive regions with significant associations between FA value and neurodevelopmental outcome measures are visible, mainly cognition at 2 years and total and manual dexterity score at school age (4–8 years). BSITD-III-NL fine motor scaled score was related to parts of the posterior-thalamic radiation and the centrum semiovale, BSITD-III-NL gross motor scaled score to parts of the CC and the posterior-thalamic radiation, MABC-2-NL catching and aiming standard score to parts of the CC, posterior-thalamic radiation and cerebellum and MABC-2-NL balance to small parts of the posterior-thalamic radiation.

## Discussion

The current study aimed to investigate the prevalence, predictors, associated brain changes and neurodevelopmental outcome of NRH in children born EPT. A prevalence of NRH in the studied population of 22.9% was found at school age (4–8 years), which is almost twofold higher than in the general population [3]. Paternal non-right-handedness and lower fine motor scores at 2 years CA were found to be statistically significantly associated with NRH. Ultimately, TBSS analysis at TEA revealed no significant relationship between brain microstructure and handedness, but did show several neurodevelopmental outcome measures to be related to white matter FA values.

The evidence of a higher prevalence of NRH in EPT children is concordant with results from previously conducted studies, as presented in a review by Domellöf et al., showing an overall prevalence of 22% in the preterm versus 12% in the term born population [3]. The more recent studies of Pascoe et al. and Burnett et al. described a prevalence of 30.9% of NRH in children born very preterm (<32 week of gestation) and 23% in EPT children, respectively [12,15]. Differences in the exact percentages observed may be due to the use of slightly different populations as well as differences in assessing handedness, as will be discussed in further sections.

Different theories to explain the higher prevalence of NRH in EPT children have been suggested. Among them are several genetic models, such as the right-shift theory, the DC model and multilocus models. The right-shift theory states that the presence of a hypothetical right-handedness gene (RS+) increases the probability of the left hemisphere becoming dominant for both language and motor skills. Since the right hand is primarily controlled by the left hemisphere, presence of the RS+ gene leads to a higher probability of developing right-handedness. Within this theory, it is suggested that preterm birth interrupts this lateralization process and thus atypical asymmetry and hand preference may develop [28]. The DC model hypothesises a combination of a “dextral allele”, which is in favour of the right hand, and a “chance allele”, which is directionally neutral. More recently, multilocus models are suggested to offer a more accurate explanation, in which the existing models can be applied to two or more (*n*) loci instead of a single locus, with all having a different combination of the hypothesised alleles [29]. However, yet no identified random loci have been found. Similarly, a timing failure of any other type of handedness gene, caused by preterm birth, may affect handedness development.

Forming a key assumption of genetic theories, parental handedness is known to influence child hand preference, as is concordant with our study results [30]. In previous studies left-handed fathers were seen more often among preterm children with a left-hand preference [31], whereas left-handed mothers were found to be more common among term born children with a left-hand preference [32]. This is in line with our findings. Interestingly, parental NRH percentages in our study group were relatively high for people belonging to the general population: 17.4% of the mothers and 18.4% of the fathers. A possible explanation may lie in the role of parental handedness or associated factors in the duration of pregnancy, although no indications for this hypothesis have been found yet. For instance, parental NRH–or the cause of it–might lead (indirectly) to factors that cause preterm birth in the parents’ offspring. In case of consistent findings in future research on this topic, this might contribute to the identification of families at risk for preterm birth [3].

A second group of theories indicates that all mixed- and left-handedness is of pathological origin and simply reflects the higher incidence of brain injury in preterm born children [3]. In this view, a familial association of left-handedness can be explained as a consequence of familial genetically linked birth complications. Furthermore, a higher vulnerability of the developing left hemisphere has been described [33]. A third explanatory model combines the two proposed lines of argumentation, by suggesting that NRH can either originate from genetic or pathological factors, with the latter accounting for the increase in NRH prevalence in preterm born children [3].

Since a study of Ross et al. found more NRH individuals among preterm children even after correcting for parental handedness, there may indeed be a reason to consider taking the hemisphere involved in the cerebral pathology into account [13]. Nevertheless, so far direct evidence of pathological NRH has been lacking [34,35]. In addition to others, we were unable to find a relationship between the presence and side of brain injury and handedness, although this might be due to insufficient power. Our results are concordant with the recent study of Pascoe et al., although their finding of an effect of cerebellar haemorrhage on handedness could not be reproduced in our cohort [12]. Exclusion of all children with at least one type of overt brain injury led to an even higher NRH percentage of 27.8% (sample size: 90), stating that other factors than overt brain injury alone play a role in the origin of NRH in this population.

On a microstructural level, the current study could not identify regions with FA values that differed significantly between different handedness groups. Since we are not aware of any other whole brain analysis study focusing on handedness, our findings add to existing literature. Pascoe et al. conducted region of interest (ROI) analysis of the CC and posterior limb of the internal capsule on DTI scans in a slightly smaller cohort of children born below 30 weeks gestation, and demonstrated a significant link between NRH and FA values of the CC as a whole, and more specifically the splenium of the CC [12]. As demonstrated, we were unable to reproduce these results with TBSS analysis in an even more preterm born cohort and did not find any of the white matter structures to be associated with NRH.

The use of whole brain analysis in the current study has some advantages compared to ROI analysis. Limitations of ROIs are its subjectivity and the fact that it cannot fully detect more global brain changes. TBSS analysis overcomes these limitations [16].

Hence, if neither overt brain injury nor microstructural brain alterations are related to NRH in EPT children, we agree with the current evidence that the brain injury theory would be insufficient to explain the higher prevalence of NRH in this population.

It is well known that both gross and fine motor development is often delayed in (extremely) preterm born infants. The observed lower scores on the MABC-2 have also been reported by others [36]. Fine motor skills at 2 years CA, were found to be less well developed in NRH children compared to right-handed children in our cohort, but an association with manual dexterity was no longer present at school age (4–8 years; [p = 0.104]). This suggests a disappearance of the effect as children grow older, but it may also be caused by using a different developmental test. Burnett et al. produced comparable results for fine motor skills [15], but Pascoe et al. did not [12]. In term born children, Freitas et al. described better manual dexterity scores in right- than left-handers (p = 0.001) [37].

We did not find an association of NRH and gross motor skills. This is in line with some other studies, although Burnett et al. found a significant difference at the expense of mixed-handers compared to their left- and right-handed peers [15,37,38]. A study in triplets did find a correlation between delayed early gross motor development (6–16 months of age) and left-handedness, but this was no longer significant when corrected for the (lower) birth weight, suggesting that birth weight rather than gestational age explains delayed motor control in left-handed children [39]. As birth weight and gestational age are strongly correlated, we tried to unravel this by including birth weight below -1 SD in the regression analyses. Even after including this covariate, fine motor skills on the BSITD-III were still significantly lower in NRH children.

We were unable to show an association between handedness and cognitive skills, which is in line with several other studies [9,40,41]. However, others did find certain cognitive domains to be linked to NRH in a preterm population [12,42], or only to mixed-handedness [15]. Since we did not reassess cognition at school age (4–8 years), it is uncertain if more clear differences may appear in later life. In term born infants, Nelson et al. also failed to find an association with general motor and cognitive skills, using the BSITD-III, but did detect an association between language and consistent (early) right-hand preference [43]. The language subtest of the BSITD-III was not performed in our cohort. Nevertheless, the term born is not entirely comparable with the preterm population, and underlying pathological mechanisms causing handedness can be different.

Interestingly, our TBSS study revealed extensive brain areas of significant associations between white matter microstructure and several neurodevelopmental outcome domains. Higher FA values, as demonstrated to be related to better outcome, are an indication of greater white matter organization [44]. Similar results have been presented for both BSITD-III scores in very preterm born children [45] and MABC-2-NL scores in very low birth weight neonates [46].

The main strength of our study is the large number of infants born EPT born who all had a state-of-the-art 3T MRI scan at TEA. The study is unique in examining the brain’s microstructure in relation to handedness in children born preterm using TBSS. It is also one of the first studies to address overt brain injury systematically assessed on MRI as possibly linked to NRH in an EPT cohort. The broad range of objectives added to the existing literature and may contribute to the understanding of handedness in EPT children.

However, the present study has several limitations. First, the relatively small sample size may have resulted in insufficient power to detect possible associations and to split up NRH into left- and mixed-handedness. This is partially due to the low incidence of EPT birth and a substantial loss to follow-up. Second, the conclusion of no association between handedness and overt brain injury, is limited by the fact that children with CP could not undergo the MABC-2-NL and were thus excluded from the study population. Another limitation is the absence of a term control population undergoing the same assessments. “Fourth, although handedness was assessed by a trained paediatric physical therapist, and the parents were asked hand preference at home (based on writing and drawing), no specific tests were done at home and the strength of the hand preference was not considered.” Lastly, even more (sub-)domains (e.g. language, behaviour, etc.) of neurodevelopmental outcome could have been assessed and/or at an even higher age. Hence, the study may have missed problems coming to light in later life or, by contrast, recovery possibilities of weaker motor skills in early life.

Existing handedness literature is surrounded by conflicting results, that may be caused by different methods of measuring handedness. Since studies vary in age of handedness assessment, settings and conditions, definition of handedness, number of observations and degree of activity complexity, our study results are not entirely comparable to those of others [47]. We obtained handedness data at school age (4–8 years), but an even higher age of assessment may be considered more appropriate, since the degree of handedness strengthens with age [24]. However, Nelson et al. state that hand preference is stable by 24 months of age [48]. Furthermore, our definition of handedness as a binomial variable is debatable. Others suggest defining handedness as a trinomial or even continuous variable, by taking into consideration the strength of handedness, or determining hand preference categories longitudinally [15,47,49].

In future, prospective studies, defining handedness as a scale (hand performance) rather than a binomial or trinomial variable (direction of hand preference) should be considered. If our MRI and DTI results can be reproduced in larger cohorts, using a standardized test, more evidence may be found of an association with brain changes and this may contribute to the understanding of aetiology of NRH in EPT children.

## Supporting information

S1 Checklist STROBE statement—checklist of items that should be included in reports of *cohort studies*. (DOCX)

## Abbreviations

NRH: Non-right-handedness
CA: Corrected age
GA: Gestational age
CC: Corpus callosum
DTI: Diffusion tensor imaging
EPT: Extremely preterm (<28 weeks gestation)
MRI: Magnetic resonance imaging
MABC-2-NL: Movement Assessment Battery for Children, second edition, Dutch version
TEA: Term-equivalent age
IVH: Intraventricular haemorrhage
PHVD: Post-haemorrhagic ventricular dilatation
PMA: Post-menstrual age
CBH: Cerebellar haemorrhage
BSITD-III-NL: Bayley Scales of Infant and Toddler Development, third edition, Dutch version
TBSS: Tract-based spatial statistics
DTI-TK: Diffusion Tensor Imaging ToolKit
FA: Fractional anisotropy
OR: Odds ratio
CI: Confidence interval
ROI: Region of interest
